# Liberating the mental health and wellbeing benefits of laughing alone: a new taxonomic model and scoping review for future research

**DOI:** 10.1007/s44192-025-00183-9

**Published:** 2025-04-28

**Authors:** Freda Gonot-Schoupinsky, Xavier Gonot-Schoupinsky, Mark Weeks

**Affiliations:** 1 School of Psychology, Faculty of Health & Wellbeing, University of Bolton, Deane Road, Bolton, BL3 5 AB UK; 2 Monaco, Monaco; 3 Institute of Liberal Arts and Sciences, Nagoya University, Furo-cho, Chikusa-ku, Nagoya, Japan

**Keywords:** Laughter, Solitary laughter, Solirisy, Solitude, Mental health and wellbeing, Scoping review

## Abstract

Laughter has been predominantly viewed as a social behavior, and laughing alone is often regarded doubtfully, even pathologized. While solitary laughter can be symptomatic of mental health issues, one motivation of this review was to explore whether it can also offer mental health benefits. Research demonstrates that over 10% of laughter occurs in solitude and there is increasing awareness that it is not only enjoyed but can serve important roles in supporting health and wellbeing. To establish a useful foundation for further exploration of its potential in enhancing health and wellbeing, this review scopes the subject across disciplines. An unprecedented search revealed 120 records (*n* = 83 academic articles *n* = 37 literary and book references) that mention solitary laughter in meaningful ways. The Solitary Laughter Model (SLM) developed for this research supported an initial classification according to the conditions in which the laughter took place be it complete solitary, public solitary, assisted solitary, or social solitary laughter. Our research reveals that laughing alone tends to be perceived as particularly problematic when done in public. Nevertheless, we also uncovered favorable mental health perspectives on solitary laughter and recent exciting clinical applications in assisted solitary laughter. The implications of these findings are discussed. The SLM is proposed as a pragmatic model to encourage precision in this nascent field of investigation and health practice. This review concludes that solitary laughter is an important human behavior deserving more scientific investigation to further unlock its mental health and wellbeing benefits.

**Supplementary Information:**

The online version contains supplementary material available at 10.1007/s44192-025-00183-9.

## Introduction

The eruption of laughter into social intercourse and its value in developing and sustaining relationships is largely beyond dispute [1]. The eruption into laughter of someone alone is a different matter. The ancient Greek philosopher Democritus is known to have indulged in solitary laughter, and though he attributed his habit to informed awareness of worldly absurdity, it inspired doubts about his mental health [2, 3]. Solitary laughter is a behavior in the shadows, often remarked for what it indicates about a person, yet we know very little about what it is and what it does. Given that laughter tends to be regarded as a social behavior [4] and strategic social signal [5], what does it mean that humans laugh alone? It matters, because solitary laughter is no mere anomaly. Research by Martin and Kuiper [6] found over 10% of laughter occurs in solitude (n = 80). Baseline data in one recent laughter intervention found 70% of participants (n = 20) reported laughing alone and 25% estimated they did so 6 times a day or more [7].

Solitary laughter matters specifically for those of us concerned with health and wellbeing for at least two reasons. On the one hand, there has in recent decades been much greater interest in the role of laughter in health and wellbeing, and with growing interest in positive aspects of solitude, solitary laughter is emerging into consciousness and practice. Dr Madan Kataria, founder of laughter yoga for health and flourishing, reports routinely laughing alone on waking and recommends it to others to render the experience of laughter deeply personal and ubiquitous in one’s life [8]. Others have been actively exploring and assessing clinical applications of solitary laughter for health and wellbeing, including Sutorius [9], Ben-Moshe [10], Mora-Ripoll [11–13], and Gonot-Schoupinsky et al. [14–16]. Ben-Moshe [10] writes, “We need to recondition our belief systems to empower ourselves to laugh alone”. By laughing alone freely, we expand the influence of laughter in our lives and positively transform the experience of solitude, potentially enhancing self-reliance and resilience [17].

On the other hand, as our review confirmed, solitary laughter has for some time been regarded as symptomatic of pathology and has been associated with diverse mental health issues, developmental disorders and other psychological problems. To buttress a social theory of laughter, the psychologist Robert Provine [18] quotes the philosopher Kierkegaard’s observation (p. 44) that one must be “a little more than queer” to do it. The problem is that this kind of perception creates fear and reluctance around the behavior that has limited its free practice for wellbeing and existential joy. Consequently, a principal aim of the present study is to investigate and stimulate interest in the application of solitary laughter for mental health and wellbeing by establishing conceptual foundations to discern and understand the different contexts in which different types of solitary laughter take place.

A first step for us was to understand that competing perceptions of solitary laughter are in part due to competing views of solitude, itself a multifaceted behavior [19]. The term “solitude” is sometimes conflated with loneliness as noted by Koch [20] (p. 29–30) and associated with depression or other negative affect. Conversely, however, it is increasingly valued as a crucial space not only for relief from social pressures but for positive self-reflection, growth, and creativity [19–23]. There are, then, both affective and cognitive elements, both causes and effects, informing the diverse ways solitude is experienced [24, 25].

Rapidly developing technology complicates solitude, and thus solitary laughter. To what degree is a person sitting alone in a forest exchanging electronic messages experiencing solitude? Or alone at home watching TV, or surfing the internet for that matter? At the same time, it has been observed that humans can experience a kind of solitude even in a room full of people [20, 23]. These complexities of modern solitude naturally impact the concept of solitary laughter. There is a need, therefore, to distinguish different types of solitary laughter, especially for those studying and applying the behavior in health and wellbeing contexts.

A key academic impediment to the study and practice of laughing alone has been an influential contention that there is virtually no truly solitary laughter, that laughter is essentially social because any laughter we experience alone is nothing more than “vicarious” or “pseudo-social” behavior. This is a hypothesis advanced most conspicuously by Provine and Fischer [26] and Provine [18] regarding laughing alone while passively viewing or reading media. Yet research and thinking around solitude has since led to the more tenable perspective that watching video and reading are not merely degraded forms of sociality but distinct forms of solitary behavior in their own right [23] even if they draw upon the social.

This insight is important because some earlier conjecture had gone further: it had dismissed solitary laughter by virtually deleting the very concept of solitude. It claimed that even laughing alone without media constitutes social behavior in effect because it is often induced through thoughts related to other people [27], or by the creation of a psychologically “split self” [28] (p. 139), allowing a person to perform for “an implicit audience” [29]. Yet “total solitude” exists [23], and the term solitude is applicable even if we are engaged in internal dialogue. There are various types and degrees of being alone [20, 23] and thus of laughing alone, with unique characteristics that require further analysis. In short, we need more precise understanding of different forms of solitary laughter to better understand and practically utilize it for health and flourishing. We attempt that in what follows.

Given our earlier stated aim of providing a foundation for further research and practice around solitary laughter, especially with regard to mental health, the research gaps we have identified are broad: there is no clear definition of solitary laughter or conceptualization of its types, and there has been no previous attempt to ascertain the scope of literature relating to the subject, including the roles solitary laughter might have in mental health and wellbeing.

Consequently, our research questions were: (1) Can we precisely define solitary laughter and conceptualize its types for practical use in mental health and other academic contexts? (2) What is the scope of our knowledge of solitary laughter? (3) Is there evidence that solitary laughter can be beneficial for mental health and wellbeing? We hypothesized, based on preliminary research, that there would be some evidence of such benefits.

The text that follows begins with definition of the key terms and introduction of our taxonomic framework before proceeding to the scoping review.

## Conceptual framework

**Fig. 1:**
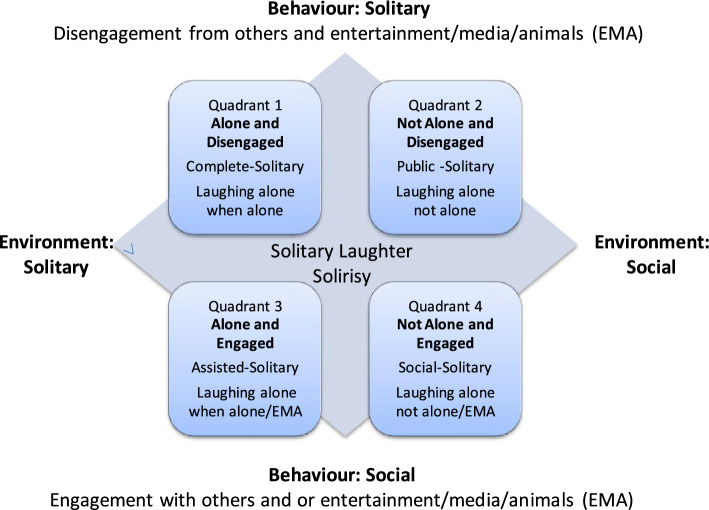
The Solitary Laughter Model (SLM). Source: Author conceived and generated

Regarding the research question about precise definition and conceptualization, the term “laughter” as we use it here refers to a physical action that may range in intensity from a barely perceived exhalation to a raucous, highly kinetic bodily eruption. Solitary laughter, we suggest, is a behavior that involves laughing alone, when alone or not, and may involve entertainment, media or animals [see Solitary Laughter Model (SLM), Fig. 1]. When it occurs in a social environment it may be distinguished from social laughter in several ways, including whether the person is clearly physically distanced within a group, is perceived to be alone with themselves, is not interacting with others, and/or by cultural understandings of normalcy. Solitary laughter can be spontaneous, provoked, or self-induced. It may be a pathological symptom. It can be produced by or result from humor or emotions or be cultivated for pleasure and/or self-care.

We created the taxonomic model below to organize data collected during the scoping review and to determine whether the formal taxonomy, the first of its kind, might furnish an effective conceptual framework for future research into solitary laughter.

### The Solitary Laughter Model (SLM)

**Fig. 2:**
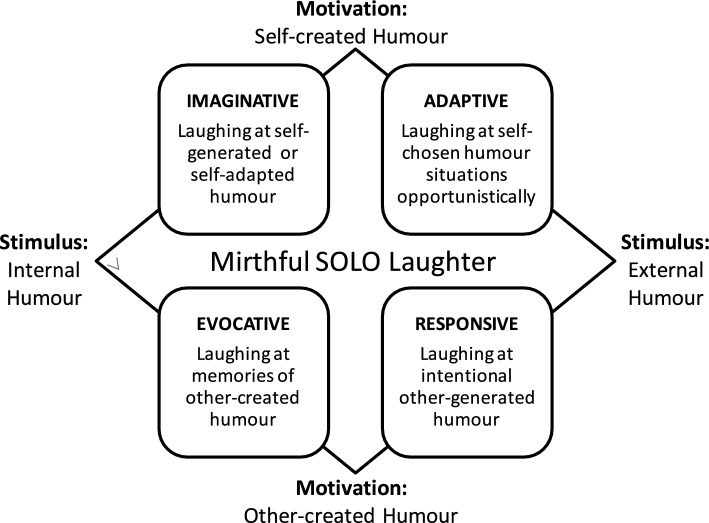
The Solitary Humour Model (SHM). Source: Author conceived and generated

The SLM, a pragmatic conceptual model we designed to support research into solitary laughter, was constructed with reference to the Solitary Humor Model (SHM) (Fig. 2), Koch [20] and Weinstein et al. [23]. The SLM allows systematic categorization of types of solitary laughter to bring greater accuracy to the examination of the subject. It was refined during this review.

Following Koch’s conceptualization of solitude and social engagement as opposite sides of a spectrum, the x-axis in Fig. 1 reflects physical detachment/engagement, while the y-axis suggests the mindset or psychological detachment/engagement. The quadrants are defined as follows, with Quadrant 1 representing the most intensely solitary laughter and Quadrant 4 the most social type of laughing alone.

Quadrant 1: Complete solitary laughter. Laughter of a person separated from others both physically and virtually in a space of relative quietude. Such solitude, though still allowing engagement with the immediate environment, is characterized by Koch [20] and Weinstein et al. [30] as typically absent of distractions and facilitative of relatively free cognition and self-reflection.

Quadrant 2: Public solitary laughter. Laughter in a public space accessible to others while socially disengaged. This is consistent with the solitude model of Weinstein et al. [23].

Quadrant 3: Assisted solitary laughter. All those instances of solitary laughter which are facilitated by some form of deliberate signifying content sourced externally to the laugher. The most obvious examples are television programs, films, much Internet content, books, magazines, which typically through humor may induce a laughter response. Less obvious examples are with family pets unless play is explicitly involved.

Quadrant 4: Social solitary laughter. Laughing alone in socially engaged contexts, which may include online real-time interactions, in which a person laughs alone among others, excluding those situations where the one making humor or a person who is the only audience of humorous content, laughs.

### A word on solitary humor

Due to its greater social conspicuousness and its forementioned contentiousness, we believe there is value in exploring the subject of solitary laughter principally in its own right, with an understanding that humor is a common source but that laughing alone may also be practiced without humor, including for therapeutic reasons.

Although enjoying humor alone is included in the Humor Styles Questionnaire (HSQ) [31] and the concept of “solitary humor” appears to be broadly understood [32], it is very rarely used. Gonot-Schoupinsky’s Solitary Humor Model (SHM; Fig. 2) recognizes this phenomenon. In the SHM, solitary humor can be thought to be differentiated into four types according to whether the stimulus is external or internal, or self-created or other-created: these are labelled imaginative, adaptive, responsive, and evocative.

## Scoping review methods

### Design

As we sought to undertake a rigorous, exploratory review investigating discourse around the concept of solitary laughter, we chose a scoping review design [33]. The Preferred Reporting Items for Systematic Reviews Scoping Review (PRISMA-ScR) checklist [34] guided review planning and analysis (see Table 1).

**Table 1:** Overview of use of the PRISMA-ScR Checklist

Section	Item^a^	Incl	Explanation if excluded
Title	1. Title	✓	
Abstract	2. Structured summary	✓	
Introduction	3. Rationale	✓	
	4. Objectives	✓	
Methods	5. Protocol and registration	✗	PROSPERO^b^ closed to ScR
	6. Eligibility criteria	✓	
	7. Information sources	✓	
	8. Search	✓	
	9. Selection of sources of evidence	✓	
	10. Data charting process	✓	
	11. Data items	✓	
	12. Critical appraisal of individual sources of evidence	✗	Not mandatory for ScR
	13. Synthesis of results	✓	
Results	14. Selection of sources of evidence	✓	
	15. Characteristics of sources of evidence	✗	Not mandatory for ScR
	16. Critical appraisal within sources of evidence	✗	Not mandatory for ScR
	17. Results of individual sources of evidence	✓	
	18. Synthesis of results	✓	
	19. Summary of evidence	✓	
	20. Limitations	✓	
	21. Conclusions	✓	
Funding	22. Funding	✓	

^a^PRISMA-ScR [34]

^b^The International prospective register of systematic reviews (PROSPERO)

### Search strategy

**Fig. 3:**
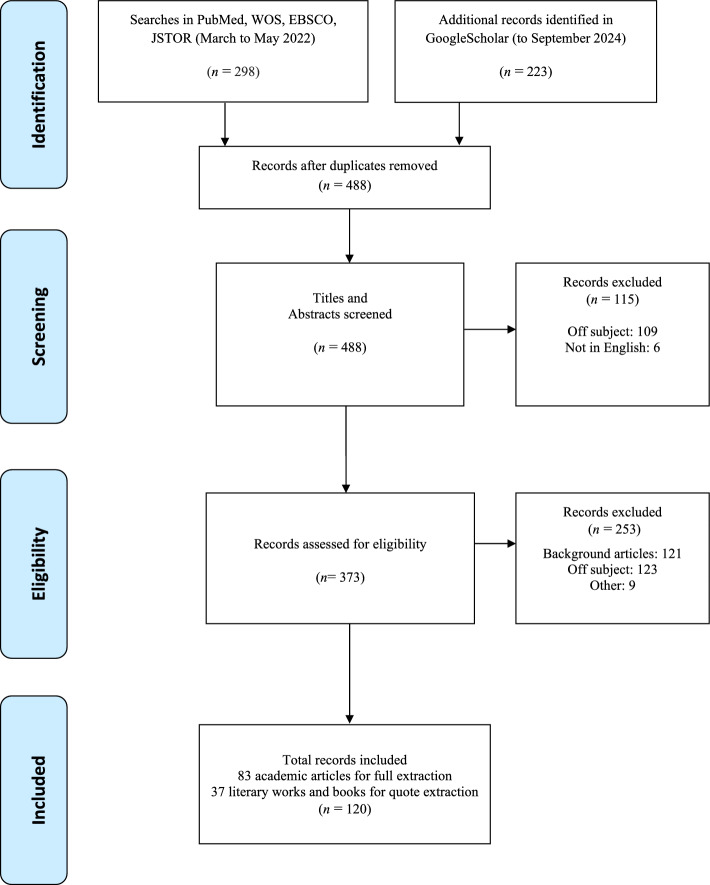
PRISMA flowchart

Database searches for articles published in English since 1970 were initially undertaken between March and May 2022, in PubMed/Medline, Web of Science (WOS) and EBSCO. Google Scholar searching was extended to September 2024. JSTOR was also searched to extend the search beyond the relatively small number of articles found in the scientific databases. While cognizant that this complicated the review, especially in terms of assessing scientific “quality,” we felt (and still feel) strongly that this served to give a broader and more balanced perspective on social as well as scientific attitudes toward solitary laughter. Search terms included “solitary laughter/laughing,” “solo laughter/laughing,” and “laugh/laughing alone”: (Solitary_laughter OR solitary_laughing OR solo_laughter OR solo_laughing OR laughing_alone OR laughing_when_alone). Use of the PICO framework [35] did not limit searching: population (all), interventions (all), comparison (none), outcome (all). Finally, complementary searches in Google Scholar were undertaken in July 2024 to broaden the scope with no restrictions on date or article type; however, for these searches only the terms “solitary” and “solo” laughter/laughing were used. Secondarily, searches for “solitary humor” and “solo humor” were also initiated but revealed virtually nothing. Searches are tracked in the PRISMA diagram (see Fig. 3), to enable transparency and replicability.

### Screening and data extraction

Articles were exported into Rayyan [36] for initial eligibility assessment, and data duplication resolution. Data extraction was collaborative, and a master Excel spreadsheet was used. Of the 488 records identified (PubMed/Medline n = 81, WOS n = 22; EBSCO n = 110; JSTOR n = 98; Google Scholar n = 177), 373 were screened for eligibility: 253 items were rejected as unsuitable (see exclusion criteria below) and 120 items were marked as potentially suitable background material.

Exclusion criteria included articles that were not in English, did not discuss solitary or solo laughter or laughing alone in the sense we use them (e.g. “alone” was used to mean “only”; solo was clearly not solitary), and where we could not access the full article. The 120 items identified for extraction included 83 academic articles and 37 literary works and books containing quotes.

### Critical assessment of articles

Article critical assessment is not mandatory within a PRISMA-ScR (see Table 1) and due to the broad range of types of records included in this review, along with the widely varying degrees to which each dealt with solitary laughter, we did not think it suitable to undertake such critical evaluation. However, each of the 120 items was analyzed to consider what type of solitary laughter was involved, and what the potential implications of the information given could be. All extractions include quotes that we attempt to interpret.

### Conceptual model to support classification of articles

The pragmatic conceptual Solitary Laughter Model (SLM, Fig. 1) was used for the classification. The previously presented SLM was inspired by another pragmatic model, the solitary humor model (SHM, Fig. 2) and by the work of Koch [20] and Weinstein et al. [23].

## Results

The Results are divided into two parts. In the first sections, we summarize, exhibit and describe the outcomes of the article extractions. These findings are supported by data shown in seven tables (Tables 2, 3, 4, 5, 6, 7, and 8). Then, in the final section, we focus on drawing out overall findings and summarize the aggregate results. The implications of these results with respect to our research questions are examined in the Discussion section. As mentioned, of the total records assessed (*n* = 120), the majority (*n* = 83) were academic articles, of which *n* = 2 are shown in Table 4 and *n* = 81 in Tables 5, 6, 7, and 8. The rest were literary works and books (*n* = 37) and quotes from these can be found in the Appendix. Academic article extractions detailed with quotes are shown in Tables 5, 6, 7, and 8 (*n* = 81).

**Table 2:** General findings as representation of solitary laughter in the academic literature

Quadrant classification (*n* = 81)^a^	Explanation	Articles (*n*)	Percentage
1. Complete solitary	SL is Principal focus	1	3%
	SL is Substantial focus	11	31%
	SL is Minor reference	24	67%
	Total within quadrant	36	100%
	Percentage of Total (*n* = 81)		44%
2. Public solitary	SL is Principal focus	0	0%
	SL is Substantial focus	2	20%
	SL is Minor reference	8	80%
	Total	10	100%
	Percentage of Total (*n* = 81)		12%
3. Assisted solitary	SL is Principal focus	2	11%
	SL is Substantial focus	11	58%
	SL is Minor reference	6	32%
	Total	19	100%
	Percentage of Total (*n* = 81)		23%
4. Social solitary	SL is Principal focus	0	0%
	SL is Substantial focus	7	44%
	SL is Minor reference	9	56%
	Total	16	100%
	Percentage of Total (*n* = 81)		20%

^a^These (*n* = 81) are the articles extracted in Tables 5, 6, 7, and 8

**Table 3:** Classification of type of laughter using the Solitary Laughter Model (SLM)

Aspect assessed (*n* = 81)^a^	Explanation	Articles (*n*)	Percentage
1. How SL is portrayed within the article: problematic, beneficial, or neutral	Beneficial	21	26%
	Neutral or unknown	28	35%
	Problematic	32	40%
	Total	81	100%
2.Degree of article focus on topic of SL	Principal focus	3	4%
	Substantial focus	27	33%
	Minor reference	51	63%
	Total	81	100%
3. Articles where SL is described in terms of pathology		13	16%
4. Articles where SL is viewed as humor-driven	Clearly Stated	35	43%
	Possible	23	28%
	Indiscernible	23	28%
	Total	81	100%
5. Articles where SL is portrayed in laughter therapy		13	16%
6. Articles where SL is portrayed in laughter meditation		5	6%
7. Articles portraying SL as difficult to undertake		21	26%

^a^These (*n* = 81) are the articles extracted in Tables 5, 6, 7, and 8

**Table 4:** Studies that track self-reported solitary laughter

Reference	Participant details	Research design	Findings
[26]	*n* = 28	Self-report using daily laughter record: 7 days	32 × higher probability of laughing socially with no media than of laughing alone with no media
	Ages 19 to 27; 13 male		5 × higher probability of laughing socially than laughing alone
			1 × higher probability of laughing alone with media than laughing socially with media
[6]	*n* = 80	Self-report using daily laughter record: 3 days	11.6% of laughter events recorded were alone
	Ages 17 to 79; 30 male		

Firstly, we consider article extractions, from the academic literature, in relation to our taxonomic model, assessing whether solitary laughter is the principal focus, substantial focus, or a minor reference in each article. Our findings (also see Table 2) reveal that of the 81 articles classified using the SLM (Fig. 1), complete solitary represented 44%, public solitary represented 12%, assisted solitary represented 23%, and social solitary represented 20%. These results clearly show that solitary laughter constitutes only a minor reference in most articles. This is why our classification was based on the extracted quote on solitary laughter (see Tables 5, 6, 7, and 8) that was most relevant to this review.

### Article classification according to the Solitary Laughter Model (SLM)

Next, we consider how solitary laughter is portrayed in each article in more depth, according to seven aspects, including whether it is portrayed as problematic, beneficial, or neutral. Our findings (also see Table 3) revealed that of the articles classified (*n* = 81): 16% (*n* = 13) related solitary laughter, solo laughter or laughing alone to pathology, 43% *(n* = 35) clearly related solitary laughter to humor, 16% (*n* = 13) to wellbeing in the form of laughter therapy, and 6% (*n* = 5) to laughter meditation.

### Article extractions

Results pertaining to article extractions gave a wide variety of insights on solitary laughter. As previously noted, 37 selected literary quotations and excerpts from books are also presented in the Appendix. Academic article extractions are summarized in five tables (Tables 4, 5, 6, 7, and 8). Empirical studies (*n* = 2) that systematically tracked laughing alone (see Table 4) revealed over 10% of self-reported laugh episodes were alone.

Article extractions from the remaining, and majority, of the academic articles (*n* = 81) led to each article being classified according to one of the Solitary Laughter Model (SLM; Fig. 1) quadrants, as summarized in Tables 5, 6, 7, and 8. Classification to one quadrant was not always easy and, as we will discuss later, can be debated. Nevertheless, it assisted with the organization and interpretation of the findings which are presented more fully in the last section of Results. And it demonstrated that solitary laughter is perceived very differently, according to each quadrant.

The findings from articles classified to Quadrant 1, Complete Solitary, (*n* = 36 as shown in Table 5), indicate wide-ranging views as to whether complete solitary laughter is negative or beneficial, or rare or frequent. However, findings from articles classified to Quadrant 2, Public Solitary, (*n* = 10 as shown in Table 6), reveal that public solitary laughter is invariably considered to be associated with mental health issues. Yet, findings from articles classified to Quadrant 3, Assisted Solitary, (*n* = 19 as shown in Table 7) indicate a positive view of solitary laughter. Finally, articles classified to Quadrant 4, Social Solitary (*n* = 16 as shown in Table 8) relate solitary laughter to social discomfort and embarrassment.

**Table 5:** SLM Quadrant 1: complete solitary laughter (*n* = 36)

Reference	Brief summary of topic	Laughter description (solitary, solo or alone)	Relevance to review	Article type and field
[37]	Examining factors around laughing and smiling in young children	*"… only 5% of laughing and smiling occurred when the child was alone."*	Shows relative infrequency of SL among young children	Observation *n* = 86 Psychology
[38]	Discusses laughter in Old English literature	*"… solitary laughter, which may be ominous…"*	SL is ominous, contrasted with social laughter in specific Old English poetry	Essay Literature
[39]	Case study of laughter professional Ros Ben-Moshe	*“There are many aspects to a solo laughter practice… As such it’s an important skill in our resilience toolbox.”*	Laughing alone is an important skill to have	Case Study Psychology
[40]	Synthesis of studies of effects of humor for older people	*"Guffaw:… strongest solitary laughter experience."*	A single, unelaborated reference to SL response in previous study	Essay: review Medicine
[41]	Sharing poetry is a valuable educational device	*"… we don't have to suffer, or struggle or laugh alone."*	Passing reference to SL as something thankfully avoidable	Essay Education
[42]	Discussion of laughter in relation to philosophies of life	*"'… because I [Miso] laugh alone'. Thus laughter constitutes an important aspect of the morals of Montaigne…"*	Refers to Montaigne on laughter, including reference to Myson's SL	Essay Philosophy
[43]	The use of satire in a political context	*"… even the most seemingly solitary laughter is “pseudosocial,” requiring a split self or imagined others…*	Solitary laughter implies a form of sociality, requiring a split self or imagined others	Essay Communication Studies
[44]	Reviews academic humor and laughter literature; presents a model for classifying them	*"Laughter is predominantly a physical behaviour, occurring alone or socially."*	Laughter alone is acknowledged and described as potentially beneficial	Scoping review Psychology
[45]	Considers some implications of laughter and humour during the COVID-19 pandemic	*“And if humour that mocks others is enjoyed alone and results in laughter and its ensuing psychophysiological benefits, then who is to judge?”*	A benefit of laughing alone at humor is that no one is hurt	Essay Positive psychology
[46]	Mentions different types of solitary laughter, including when alone	*"There are many cases of spontaneous laughter in older people when alone, and even when their thoughts are not bent upon anything peculiarly witty… children laugh aloud when they are alone because they are glad the world is so beautiful…”*	Solitary laughter exists and may be simply joy	Questionnaire (*n* = 700 plus) Psychology
[47]	Examines the differentiation of expression and social negotiation	*"… spontaneous and solitary laughter are primarily expressive, the ingratiating smile primarily negotiating."*	Solitary and spontaneous laughter identified as primarily expressive	Essay Psychology
[48]	Autoethnographic account of laughter professional Joe Hoare	*“…solitary laughter is essential…The choice of intentional solitary laughter for joy is life-affirming as it is an act of self-empowerment.”*	Laughter professional recommends solitary laughter	Autoethnographic account Psychology
[49]	Case study of a schizophrenic hoarder	*"He had strange behavior such as separating himself from others, speaking and laughing alone, and collecting rubbish."*	Case study subject is diagnosed in part through SL	Intervention (*n* = 1) Psychology
[8]	Case study of Dr Madan Kataria, creator of Laughter Yoga	*“I begin my day at 4am with laughter… you can practice laughter anywhere, anytime. So, you don’t have to depend on somebody else…”*	Creator of Laughter Yoga recommends solitary laughter	Case study Psychology
[50]	Studies factors in laughter among preschool-age children	*"… children seem to laugh very seldom alone…"*	6.3% of children's laughter was solitary, using toys but no media	Observation Psychology
[51]	Information technology is described as threatening to human community	*"But we don’t laugh alone; laughter at something is always an invitation to laugh with someone."*	Broad assertion that humans do not truly laugh alone	Essay Philosophy
[52]	Analysis of concepts of the human and gender in Ancient Greek myth	*".. the god’s solitary laughter, a laugh that emerges from his alienation from himself and the world of men…"*	Ancient acknowledgement of humorously self-induced solitary laughter and connection to self-alienation	Essay Philosophy
[53]	Architectural arrangement study includes human comical organization and laughter	*"… it is difficult to laugh alone; it is easier to laugh in a crowd, which establishes in laughter a social meaning."*	Marginalizes SL while referencing Bergson's social theory of laughter	Essay Architecture
[54]	Reviews studies of humor in terms of communication	*"… Solitary laughter, compared to laughter among interlocutors, is rare…"*	Solitary laughter is noted for its rarity, justifying a focus on social, communication theories	Essay: review Communication studies
[55]	Infants developing humor awareness with parents	*"Children with autism are more likely to exhibit solitary laughter…"*	Suggests social nature of laughter is challenged by autism	Experiment *n* = 20 Psychology
[56]	Studies the development of infant humor	*"… they laugh when alone in response to stimuli that do not typically evoke laughter."*	SL is seen as more common in children with autism	Observation *n* = 37 Psychology
[11]	Describes therapeutic techniques using laughter	*“’intense laughing out loud’, can be experienced either alone or in groups…”*	Laughing alone is recommended	Review Lifestyle Medicine
[12]	Therapeutic simulated solo laughter is referred to	*"… further exercises can be developed and added: to laugh alone, in pairs or in a group…"*	Therapeutic simulated solitary laughter is referred to in several contexts	Narrative review Lifestyle Medicine
[13]	Therapeutic use of simulated laughter alone and in groups	*"Solo laughter refers to a series of different simulated laughter exercises for laughing alone."*	Solitary laughter is offered as a therapy option, and referred to as “solo laughter”	Essay Lifestyle Medicine
[57]	Explores the potential for using psychology in military contexts	*"He laughs where he would never laugh alone…"*	Peer pressure facilitates laughter in a crowd in a way not possible when alone	Essay Psychology
[58]	Case study of laughter therapy leader	*"… the ability to learn to laugh alone is critical to our wellbeing and sanity."*	SL is important for wellbeing, even if considered unusual	Case study Mental Health
[59]	Solitary play gestures in animals noted. SL is seen in context of vicarious sociality	*"Like other great apes, humans not only smile in social contexts but also smile when alone."*	Despite evidence animals laugh alone, the vicarious sociality hypothesis regarding solitary laughter is stated	Essay Biology
[60]	Examines factors affecting laughter in speech situations	*"The few examples of solitary laughter may be responses to imagined or recalled social encounters…"*	Introduction only cites previous studies asserting SL is uncommon and pseudosocial	Observation Psychology
[61]	Critical interpretation of Bergson's theory of the comic	*"Bergson's silencing of laughter 'among solitary subjects' seems to serve another master…"*	Critical analysis of the treatment of laughter as essentially social	Essay Philosophy
[62]	Examines the adaptation of a literary work to the screen	*“the duplicity of Gérard Philippe's Julien had been conveyed…through…mechanical movements, shifting eyes, and solitary laughter.”*	Solitary laughter is associated, along with other behaviors of a character, with dishonesty	Essay Literature/film
[9]	Laughing meditation is described as a therapeutic technique	*"The laughing meditation…. can be done alone or with the 'other' in the mirror or with any group of participants."*	A 15 min "laughter meditation" can be performed alone to produce relaxation and a feeling of "wholeness"	Essay Therapy
[63]	Argues the positive developmental, social effects of communal laughter	*"…solitary laughter is most likely secondary to the possibility of laughing together…"*	Single remark subordinates SL to social laughter	Essay Education
[64]	Argues the importance of corporeality in human relations	*"… laughter seems to be essentially communal; solitary laughter always feels somehow derivative."*	Claims solitary laughter is not genuinely spontaneous	Essay Education
[65]	Describes four social functions of humor	*"… we can readily understand why one does not laugh alone."*	Dismissal of solitary laughter despite acknowledging it with media and through memory	Essay Sociology
[66]	Examines the notion of dissolved selfhood in laughter	*"Incongruity theory also alleviates the problem posed by solitary laughter to theoretical approaches focused on a communicative function…"*	Solitary laughter is difficult to accommodate to social theories of humor-driven laughter	Essay Philosophy
[67]	Following a preliminary survey, the general dismissal of solitary laughter by researchers is questioned	*“The vast majority of respondents laughed alone: Why has there been such a vigorous defense of an essential sociality in laughter solitude?”*	Solitary laughter is a frequent behavior that merits attention	Essay with questionnaire-based field study (*n* = 104) Philosophy

**Table 6:** SLM Quadrant 2: public solitary laughter (*n* = 10)

Reference	Brief summary of topic	Laughter description (solitary, solo or alone)	Relevance to review	Article type and field
[68]	Historical writing about immigration	*"… shared in a hearty, departing laugh with himself…"*	Describes a person laughing in public as a disengaged viewer	Essay History
[69]	Description of T'ang culture	*"… describes such women as those who'… speak alone, laugh alone, melancholy-minded and distracted'."*	SL by women associated with severe psychic disorder, melancholy, evil in Tang Dynasty	Essay History
[70]	Case study of a hebephrenic schizophrenic who sometimes engages in solitary laughter	*"Although he is basically withdrawn and sedentary, he sometimes shows solitary laughter and mumbling…"*	Solitary laughter is described in a schizophrenic	Case study (*n* = 1) Psychiatry
[71]	Assesses community knowledge of mental health issues in Oromia Ethiopia	*"Talking or laughing alone and showing strange or unusual behaviors were described as symptoms of mental illness by the majority."*	Talking or laughing alone are associated with mental illness	Survey (*n* = 420) Medicine
[72]	Assesses health extension workers’ (HEWs) knowledge of mental health issues in Jimma Ethiopia	*"Almost all (240, 92.7%) of the respondents reported talking or laughing alone as a manifestation of mental illness."*	Talking or laughing alone are associated with mental illness	Survey (*n* = 259) Medicine
[73]	Case of 37-year-old woman whose symptoms were relieved with Vitamin B12	*“the patient was seen laughing alone in her hospital room….”*	Laughing alone is described as a symptom of Vitamin B12 deficiency	Case Study Medicine
[74]	Assesses community perceptions of mental health issues in Vietnam	*"Frequently mentioned symptoms of mental illness were talking nonsense, talking/laughing alone and wandering."*	Talking or laughing alone are associated with mental illness	Cross-sectional (*n* = 200) Medicine
[17]	Case study contributor recounts the solitary laughter of a passer-by and its impact on him	*“… it helped bring laughter into the domain of solitude, for me… it further enhanced my times alone.”*	Laughing alone in public can be transformative	Autoethnographic case study
[75]	SL is associated with schizophrenia	*"He exhibited frequent mumbling and solitary laughter, both ranging in volume from soft and barely audible to loud and disruptive."*	SL identified in a schizophrenic patient as a behavior to be modified	Intervention (*n* = 2) Psychology
[76]	Assesses family beliefs towards mental health in Kano, Nigeria	*"The symptom cluster ‘unconscious behavior’ was most often associated with strange behavior, talking or laughing alone, improper dressing…"*	Talking and laughing alone is mentioned as a symptom of mental illness by most (90.5%)	Survey/interview (*n* = 266) Medicine

**Table 7:** SLM Quadrant 3: assisted solitary laughter (*n* = 19)

Reference	Brief summary of topic	Laughter description (solitary, solo or alone)	Relevance to review	Article type and field
[77]	Studies the effect of social context on laughter, smiling in children watching videos	*"These showed that children laughed significantly more in pairs than alone… and in groups than alone…"*	Shows that laughter is increased by social context when watching videos	Cross-sectional (*n* = 20) Psychology
[32]	Seeks insights from a humor scholar on mental health benefits of humor and mental health	*"So 'solitary humor' is effective to the degree that it is actually funny."*	Health benefits associated with solitary laughter, especially with media	Case study (*n* = 1) Psychology
[78]	Discusses ethical issues around use of media laugh tracks	*"No one likes to laugh alone…"*	Discomfort with solitary laughter is given as a key reason for TV laugh tracks	Essay Sociology
[79]	Describes the cultural contribution of a radio host	*…"the laugh track… was intended to make people listening at home feel as if they were not laughing alone…"*	Canned laughter may be used to alleviate difficulty of solitary laughter	Essay Cultural studies
[80]	Investigates whether sharing humor enhances expressive laughter response and humor rating	*"When young children are listening to laughter-provoking material, they laugh more when with a companion than when alone."*	Humorous laughter was facilitated with company	Intervention (*n* = 70) Psychology
[81]	Compares laughter time when watching videos alone, or with others	*"Provine reported that laughing was more than 30 times as likely to be performed by research participants in social than in solitary settings…"*	Frequency and time spent laughing were significantly less in the alone condition	Intervention (*n* = 162) Psychology
[14]	Prescribes laughter, including solitary laughter, with the aid of a smartphone	*"… participants related their solo laughter to health, happiness, humour, and self-discovery."*	A trial using recording of one's own laughter to stimulate regular laughter for wellbeing	Intervention (*n* = 21) Psychology
[82]	Protocol for study using recording to facilitate simulated solitary laughter	*"The Laughie was created as a quick and convenient way to laugh alone…"*	Use of recorded laughter to facilitate solitary laughter	Feasibility study protocol (*n* = 40 planned) Psychology
[83]	Presents hypotheses for the laughter response based on neurology	*"… the fact remains that we sometimes do laugh when we are alone…"*	Acknowledges that we do laugh alone, specifically with media	Essay Psychology
[7]	Pilot study of a Group Laughie prescription that recommends also laughing alone	*“… participants were prescribed to laugh with the recording alone or with people of their choice.”*	Solitary laughter can be a beneficial addition to laughter prescriptions	Pilot study (n = 20) Nursing
[84]	Instructions for a Group Laughie prescription, with instructions to also laugh alone	*“.. individuals come together to create a group laughter recording, which they are then prescribed to laugh along with alone or with others”*	Laughing alone can be integrated into laughter prescriptions	Commentary Nursing
[85]	Surveys preferred methods of receiving information	*"… with TV…. if there is something funny you end up laughing alone"*	Because TV is not interactive, it does not allow laughter to be shared, which is considered a negative	Focus groups (*n* = 55) Information Science
[86]	Attempts methodical study of factors around comic experience and laughter using media	*"Among the reasons are…'foolish to laugh alone'…"*	Evidence that solitary laughter may be considered foolish	Cross-sectional (*n* = 60) Psychology
[87]	Ten trials observing students’ reaction to a laugh stimulus finds most laughed in the early trials, but their laughter declined later	*“Canned laughter is also an effective solution to the surprisingly difficult task of evoking the social behavior of laughter in the laboratory.”*	Ten consecutive 1-min trials, each consisting of an 18-s sample of laughter were used	Intervention (*n* = 128) Psychology
[88]	Studies the effects on empathy of laughing with comedy	*"Solitary laughing at a [situational comedy] has a significant influence on the emotional interest in others."*	Solitary laughter shown to benefit interaction with others and the world	Cross-sectional experiment (*n* = 44) Psychology
[89]	Tests whether occupying a position of 'low power' increases laughter	*"… participants laughed alone because they had an audience in mind…"*	Finds support for the "implicit sociality" theory of solitary laughter	Intervention: (*n* = 32/97) Psychology
[90]	Compares effect of laughing with others and laughing alone with media on functional disability risk	*"Laughing in a conversation with friends reduced the risk of functional disability by approximately 30% compared to laughing alone."*	Laughing with friends reduced the risk of functional disability by 30% compared to laughing alone with media	Prospective cohort (*n* = 12,571) Medicine
[91]	Examines the relationship between laughter and dyspepsia	*"… to investigate possible associations between… social laughter versus solitary laughter, and FD [functional dyspepsia]."*	Solitary laughter with media showed some positive correlation with dyspepsia	Survey (*n* = 8923) Psychology
[92]	Compares solitary emotional responses in autistic and typical adolescents when watching videos	*"Research shows that TD [typically developing] children laugh more frequently when engaged in a shared activity than when they experience the activity alone."*	ASD adolescents produced significantly more laughter when alone watching videos than those "typically developing."	Intervention (*n* = 40) Psychology

**Table 8:** SLM Quadrant 4: social solitary laughter (*n* = 16)

Reference	Brief summary of topic	Laughter description (solitary, solo or alone)	Relevance to review	Article type and field
[93]	Explorative study of the practices used bv pilots to correct various problems during flights	*"The COP’s (copilot) following solo laughter seems to acknowledge and handle his embarrassment over his faulty action (giving incorrect coordinates…"*	The term solo laughter is used once, in the context of embarrassment in aircraft pilot interaction	Qualitative research (60 h of recorded data) Sociology
[94]	Observed laughing and crying in preschool children	*"No record of freeplay was made unless at least four children were present… Children laughed most frequently when associated with other children or adults…"*	Relatively high level of solitary laughter (15.5%, p121), although the author nevertheless concludes that laughter is highly social	Cross-sectional observation study (*n* = 29) Psychology
[95]	Learning disabled children participated in a problem-solving task requiring group decision making	*"…learning disabled males… increase(d) their laugh-alone behaviour in the 'Pep Talk' condition; all other groups tended to reduce their solitary laughing…"*	Solitary laughing mentioned once. Learning disabled males seemed "discrepant" in their propensity to laugh alone	Experiment (*n* = 110) Education
[96]	An orthography for recording laughter in which American and Zotzil laughter is examined	*"…to laugh alone (or be sole non-laugher) in a group is a form of temporary ostracism with immediately personal significance"*	Laughing alone is seen as rare and associated with ostracism	Analysis of recordings. Anthropology/Linguistics
[97]	Discusses interactions in conversational space	*"Laughing alone cannot avoid mockery, and it inevitably remains pompous."*	Laughing alone during conversation is considered to contain ridicule	Essay Sociology
[98]	Laughter and collective awareness: The cinema auditorium as public space	*"Solitary laughter can create a peculiar imbalance or asymmetry";"…in the cinema solitary laughter needs confirmation"*	The term solitary laughter is used twice; it is seen as disruptive, unsettling, needing redress	Essay Communication Studies
[99]	Explores the Taboo-Repression-Denial Hypothesis; embarrassment is viewed as intrinsic to social interaction	*"… the subject's greater tendency… to laugh alone in talk about embarrassment";"the interviewer laughs alone more frequently in non-embarrassment talk"*	The "laughing alone" of both "subject" and "interviewer" is associated with embarrassment	Verbal discourse analysis (*n* = 10); 20 laughter exchanges Sociology
[100]	Examines laughter in the context of a funhouse (house of mirrors)	*"… the laugher, although alone, pays tribute to the necessity of copresence by attempting to construct a social environment to sustain his or her laughter."*	Solitary laughter is hypothesized to be implicitly calling others	Observation Sociology
[101]	Analyses plays, focusing on the play and meme “Women Laughing Alone with Salad.”	*“Sheila Callaghan’s Women Laughing Alone with Salad (2015) serves as a touchstone for analysing performances of ludic feminist futility in plays by diverse writers…”*	Suggests the meme of women laughing alone with salad reflects a form of feminist absurdism	Essay Theatre Studies
[102]	Discusses use of dance to foster social skills in children with developmental issues	*"He also displayed temper tantrums and laughing alone."*	Laughing alone is seen to suggest developmental delay in a 6-year-old child	Intervention (*n* = 10) Education
[103]	Mentions laughter in relation to courtly etiquette in the thirteenth century	*“Laughter is thus understood as a social code…. Hence any solitary laughter is strictly banned: Ne rit pas seul, car c’est folie.”*	An example of proscription of solitary laughter in medieval society in France	Essay Theatre Studies
[104]	Assesses solitary laughter in discourse between speakers and listeners	*“Solitary speaker laughter would seem to be more declarative than invitational… The significance of solitary listener laughter is less clear.”*	The utility of discourse solitary laughter is still not well understood	Experimental research using 55 dyads Psychology
[105]	Examines standup comedy in social context	*"… people are justifiably nervous about laughing alone and what that might reveal…"*	Fear of laughing alone in public is observed in a performance context	Essay Cultural Studies
[106]	Observes sharing of humor and laughter in children with autism or Down's syndrome	*"The relative frequency of solitary and non-shared laughter was significantly higher in the autism group…"*	Typology of solitary laughter is described after observing children	Cross-sectional (*n* = 35) Psychology
[107]	Interprets a Shakespearean dramatic character	*"No one in company-and as little at the theater-likes laughing alone or not being able to laugh when the others do."*	Notes discomfort in unshared laughter, or not laughing when others do	Essay Theatre Studies
[108]	Systematically reviews touchscreen interventions to support wellbeing in those with dementia and caregivers	*“…solitary laughter tended to occur when the person with dementia was lost for words, perhaps in an effort to manage uncomfortable feelings.”*	Laughing alone is associated with social discomfort	Systematic review (*n* = 16) Psychology

### Summary of overall results

Overall, solitary laughter constitutes only a minor reference in most articles (see Table 2). The generation of solitary laughter from humor was identified in 78% of articles, either clearly (43%) or possibly (28%) (see Table 3). None of the articles give details on frequencies of such behavior, but the high percentage of texts referring to this subject suggests that solitary laughter is very often associated with humor. Only two papers (see Table 4) focused on empirically tracking laughter undertaken alone.

Among the reviewed articles, solitary laughter is described explicitly as problematic in 40%, including 16% pathologizing the behavior (see Table 3). Almost all articles that referred to public solitary laughter (Table 6) related it to negative perceptions of mental health. Solitary laughter was associated with beneficial effects in 26% and therapeutic effects in 16% of articles.

Regarding mental health practices, the earliest reported use of solitary laughter among the articles reviewed was Sutorius’s [9] laughter meditation. Later reports include those from Mora-Ripoll [11–13], and Kataria et al. [8] and reveal solitary laughter employed as an adjunct to the use of laughter in social therapeutic contexts, extending the therapeutic practice of laughter into the private domain. It is also referred to in relation to recovery from cancer [39]. Several books [10, 16, 109] discuss the mental health benefits of laughing alone in more detail. Empirical evidence as to the mental health benefits of daily laughter, explicitly including laughing alone, is found in studies using the one-minute Laughie Laughter Prescription developed in 2017 by Gonot-Schoupinsky [14, 16]. The Laughie emphasizes the importance of laughing alone (although it can also be undertaken with others and in groups). In one study, participant wellbeing levels, as measured by the WHO (5) Wellbeing Index, increased by 16% post-intervention and a range of mental health benefits were identified, including anger reduction, increased resilience, relaxation, a more positive outlook, sleep improvement and reduced anxiety [14].

The following is a summary of results relating specifically to the 4 categories of the Solitary Laughter Model (SML).

Complete solitary laughter (Table 5), which we define as laughter of a person separated from others both physically and virtually in a space of relative quietude, was a subject of 44% of academic articles reviewed, making it the most heavily represented of the four types. Specific instances identified in the literature were: spontaneous reaction to an object or event perceived as humorous in the surrounding environment; remembering a humorous event or communication; reflecting with humor upon a previous event while alone; imaginatively conceiving humor through creative cogitation. Complete solitary laughter may also occur without readily identifiable humor and has been identified with experiences of novelty and joy. Deliberate self-induced laughing alone without recourse to humor has been described as a technique for extending therapeutic group laughter beyond the communal setting [11–13].

Public solitary laughter (Table 6) refers to cases of solitary laughter in which others may see the person laughing. Although it is a socially conspicuous laughter, it was referred to in only 11% of articles and there were no articles principally focused on this subject. Yet, of the academic texts that we placed here, all but one alluded to some perception of non-normative behavior, including diagnosed schizophrenia [75] and autism [106]. Community surveys found solitary laughter to be considered a primary indicator of mental illness [71, 72, 74, 76]. In short, as it appears in the literature, public solitary laughter is a particularly problematic kind of solitary laughter.

The assisted solitary laughter category (Table 7) contains all those instances of laughter facilitated by some form of deliberate signifying content sourced externally to the laugher, as with television programs, films, Internet content, books, magazines. It was referred to in 24% of the articles, and it was the principal (12%) or a substantial (53%) subject of 65% of those. It appears to be a common means of laughing without others present in response to humor. The role of “laughter tracks,” so-called “canned laughter,” in stimulating laughter and thus supporting the laughter response in those who may be removed from social context is well documented [78, 79]. However, not all assisted laughter is attributed to humor, and this is especially relevant to laughter therapy. In therapeutic contexts, laughing alone using the Laughie (Laugh Intentionally Everyday) laughter prescription can be defined as assisted solitary laughter, as it uses a smartphone to help induce and sustain laughter [14, 16].

Social solitary laughter (Table 8) refers to laughing alone in social contexts and is distinguished from public solitary laughter in designating solitary laughter during *direct social interaction* that does not reflect engagement with the interaction. Social solitary laughter was referred to in 21% of articles, though it was the principal focus of none and a minor reference in 60%. Our review found that this kind of unselfconscious solitary social laughter was identified in social psychology with learning disabilities [95], developmental issues [102] and autism [106].

## Discussion

The following discussion based on the above results is organized sequentially as responses to the three research questions set out in the Introduction.

### Research Question 1): Can we precisely define solitary laughter and conceptualize its types for practical use in mental health and other academic contexts?

The formal definition of the term “solitary laughter” proposed at the outset provided a useful foundation for our work, encompassing all types of laughing alone that we and others have identified. A major challenge we faced in utilizing and refining our Solitary Laughter Model (SLM) was that well over half of the academic articles contained only brief and somewhat marginal references to solitary laughter. The next was understanding what kind of solitary laughter or laughing alone was being referred to in those references. Consequently, a good deal of close reading of the texts to establish the context, and from that the implicit type(s) of solitary laughter, was required. Even then, some cases necessitated educated guesses. For these reasons, we can report that on the one hand classifying references to solitary laughter in existing texts was difficult, and on the other hand, that this confirmed the need for a model such as the SLM to bring more precision to future studies involving the subject of solitary laughter and humor.

Most importantly, the SLM proved effective because it was able to accommodate the vast variety of instances of solitary laughter discovered in the review. It was also able to facilitate critically nuanced distinctions and analysis. For example, as mentioned in the Introduction, assisted solitary laughter is a crucially contested area regarding its status: is it solitary or social behavior? For Provine [18] and other scholars such as Martin and Kuiper [6] who posit a sweeping social theory of laughter it is “essentially” the latter, though this is contested by Weeks [67] and Prusak [61]. Moreover, the social effect is not all one-way, since Weeks [67] found some respondents reported laughing more readily alone than with others.

The review suggests that laughter alone with passive media assistance is different to the completely solitary form, but we did not find empirical evidence supporting simple conflation with social behavior. The category of assisted solitary laughter in the SLM acknowledges something that activates unique pleasures and psychological processes. Our point is that a simple social/solitary binary is inadequate for understanding how laughter functions in various contexts, and this has probably hindered the study and practice of certain types of laughter in relation to mental health. The SLM allows us to move beyond that oversimplification to explore the complexity of how we use laughter, better understand related pathologies, and activate the use of solitary laughter in therapeutic contexts.

### Research Question 2): What is the scope of our knowledge of solitary laughter?

Our study confirms solitary laughter has received remarkably little academic attention, yet it also reveals that solitary laughter is a significant human behavior. Empirical research (Table 4) dating back over 20 years shows that more than 10% of laughter episodes can occur when we are alone and that it can be generated in various ways.

As previously noted, the medical and psychological literature tended to identify it as a marker of some form of non-normative, possibly pathological, behavior related to cognitive, learning and socialization issues. The SLM classification revealed that solitary laughter is viewed particularly negatively when it is undertaken in public. Seeing someone laughing alone can be alarming as we know that laughter can be a symptom of pathology or related to mental health problems such as psychotic episodes. Merchán-del-Hierro et al. [110] relate that “laughter may be a medical phenomenon in many neurologic and psychiatric entities, including pseudobulbar affect, tics, hallucinations, psychogenic disorders, stereotypes, and seizures.”

However, an important point arising from the classification of the data on solitary laughter into types using the SLM is that we could see that it is not solitary laughter in general but specific manifestations of solitary laughter that are subject to negative perceptions. Laughing alone with the aid of media, for example, appears to be so pervasive as to be “normal.” Laughing completely alone appears to become problematical when viewed from outside; that is, when the solitude is witnessed by an observer who may then pass judgment on the behavior. Interestingly, there is relatively little evidence that solitary laughter outside a public or social context is experienced as a problem by the one laughing. This suggests that when it does evoke embarrassment it is because the laugher is at that moment viewing themselves from outside themselves, a notionally public perspective.

We should point out that our review showed solitary laughter has not always been pathologized within psychology. Firstly, early psychologists such as Freud and Hall remarked positive functions of the behavior related to enjoyment, joy and gratitude, Freud observing that “*…one can enjoy the comic alone… I can laugh heartily over it alone*” [111] (Freud, 1960, p. 143; Appendix). Beginning in the late Twentieth Century, there have emerged more references and increasingly closer attention to benefits of laughing alone, leading to formal mental health practices. The Humor Styles Questionnaire developed by psychologist and humor specialist Martin and others includes the use of humor alone as a type of “self-enhancing humor” [31] (Martin et al., 2003).

The ability and desire to enjoy and even generate humor alone has received relatively little scientific attention, but our review indicates that humor is a common, though not exclusive, source of solitary laughter. In that regard, we want to mention the useful supplementary data gleaned from our investigation of non-scientific texts in this study (Appendix). When Martin et al. refer to the use of “self-enhancing humor” to address “absurdities of life” [31] they are drawing not only on psychological science, but also on a much longer history of philosophy. Our supplementary exploration of non-scientific texts revealed that philosophical figures, including Augustine, Montaigne, Schopenhauer, and Nietzsche, as well as literary figures such as Shakespeare,Tolstoy, Wordsworth and Gibran (all quoted in the Appendix), have long recognized that humor and laughter may be induced when contemplating in solitude upon life, the world we live in, and the apparent conceptual dissonances that emerge. Moreover, the solitariness is seen to grant a special value to the behavior, so that it has been condoned for centuries, including, as Gilhus [112] points out, in monastic communities. We are not offering these texts as compelling evidence for the value of the behavior in fostering mental health (hence, the inclusion of the data as an Appendix) but wish to acknowledge that such non-scientific texts have been valuable in marking unexplored territories for us to explore further.

### Research Question 3): Is there evidence that solitary laughter can be beneficial for mental health and wellbeing?

It was noted in the Results section that solitary laughter was associated with beneficial effects in 26% and therapy in 16% of articles. Most of these articles were recent, reflecting the fact that this is a nascent field of research and practice. The anecdotal evidence and available empirical data from these studies, including the applied Laughie Laughter Prescription [14, 16], indicate that laughing alone merits encouragement, as it can be an enjoyable and beneficial experience, and one that has a range of mental health benefits, including improved mood, energy and relaxation. Our hypothesis that solitary laughter can have beneficial effects on mental health appears therefore to be supported by modest evidence.

As a new therapeutic practice, despite a dearth of empirical data, initial findings that solitary laughter can support mental health are therefore encouraging. However, more research is needed. We also recommend that mental health professionals investigate laughter therapy themselves to support their patients in enhancing wellbeing, autonomy and resilience.

These recommended therapeutic uses of laughter often do not rely on external humor to instigate laughter but seek to derive psychophysiological benefits from the laughter itself. Laughter is seen as an exercise that provides some of the physiological benefits of exercise and the mood-enhancing benefits of laughter to promote overall wellbeing. Laughter therapy that is humor-induced (for example, by using McGraw’s 7 habits of humor, as in work undertaken by Ruch et al. [113]) tends not to explicitly highlight the therapeutic and practical benefits associated with laughing alone, and therefore we have not addressed it in this review. This reticence to highlight solitary laughter in the therapeutic domain, both as an integral part of the therapy and as a term itself (Mora-Ripoll has also used the term “solo laughter”), is thought provoking. We hope we have made a clear case for the term solitary laughter. However, in recognition that the term may have a negative connotation for some, “solirisy,” from “solo” and the Latin for laughter (risus) and inspired by the term soliloquy has been proposed [114].

## Limitations

Our research has several limitations. Firstly, it was conducted in English and was therefore somewhat limited in its capacity to uncover potentially unique, culturally specific issues around the subject. Secondly, our work is mainly theoretical in nature, therefore the Solitary Laughter Model (SLM), and our definition of solitary laughter, would need to be tested by others and potentially refined. One limitation of using the SLM to classify laughter is that substantial information is needed to do this correctly. As there was not always sufficiently detailed information within the texts, our classification was not always conclusive. It is also difficult to distinguish whether humor is involved in the laughter in some circumstances, which led us to use the classification “indiscernible” in Table 3. Furthermore, the SLM as it stands does not provide detailed information on laughter types or mental health issues that might be implicated. Finally, due to the remarkably scant research in this area, we have undertaken a broad scope that includes texts that do not lend themselves to critical assessment, and therefore our findings would need to be reviewed when higher quality evidence becomes available. This review is not presented principally as a summary of scientific evidence. Rather, it is a broad scoping review that attempts to bring together diverse insights into solitary laughter as a foundation for further study, including scientific analyses, in this new area.

## Future research

Concerning the Solitary Laughter Model (SLM) introduced here, we hope to advance its use and continue to refine it based on feedback and applications to serve study and practices in mental health, as well as in other fields. Indeed, further models will need to be added to investigate this field more thoroughly and gain insight into the different types and qualities of solitary laughter.

There are many avenues to follow to gain more insight into solitary laughter, including its frequency, perhaps using measuring devices which are suitable to track laughter in everyday life [115] since laughter frequency can vary across cultures [116] and, importantly, research has also shown that self-reported laughter may not be accurate [117]. The quality and type needs to also be investigated, as laughter can vary widely, including due to conditions such as autism [118]. We don’t have much insight into pathological or abnormal laughter being undertaken alone, as it is mainly observed behavior; therefore, this also requires investigation and would benefit from application of the SLM. We also need more investigation of specific triggers of each of the four types of solitary laughter proposed in the model, perceptions of each type and the impact of those perceptions on solitary behavior.

Another useful line of enquiry is to continue to explore the unique mental health benefits of laughing alone, since these are unlikely to be identical to socially engaged laughter. Most obviously, pleasure derived from laughing alone could make solitude more palatable to those who are alone involuntarily through social, life circumstances. But it could also allow people to practice humor and laughter more freely, with less concern for social censorship. Furthermore, we also believe strongly that we should explore how solitary laughter can serve, like voluntary solitude in general, to support the development of healthy autonomy and personal creativity [20, 21], including the creation of one’s own humor. In other words, solitary laughter, if given due attention, has exciting potential to both extend the practice of laughter for life enhancement in areas such as self-reliance, resilience and joy, and to positively transform the experience of solitude for everyone [17].

There are many areas associated with self-care that should be productive for future research and practice, especially in conjunction with positive psychology and the increasing study of solitude in general. We believe the present study will facilitate that, especially the Solitary Laughter Model (SLM), which will allow greater precision in the analysis and deployment of laughing alone to bring it out of the shadows. Finally, and perhaps most importantly, we would encourage further expansion of explorative clinical activities and studies already being undertaken, including programs that prescribe and monitor interventions using solitary laughter practices.

## Conclusions

Solitary laughter is a complex behavior that is not well understood or documented. For this reason, we seek to foster interest and insight into the behavior to help psychologists and others working in the mental health field to begin to interpret solitary laughter and apply it therapeutically. Despite empirical research pointing to more than 10% of laughter taking place alone, the behavior is often overlooked, partly due to outdated concepts of solitude. Using contemporary understandings of solitude, we propose that this multifaceted behavior has been underestimated. Our scoping review used 120 records (83 academic articles; 37 literary works and books) to draw out insight into the concept of solitary laughter and its types. Our taxonomic solitary laughter model (SLM) provides an initial conceptual framework. It distinguishes solitary laughter according to four types: (1) complete solitary, (2) public solitary, (3) assisted solitary, (4) social solitary laughter. These “types” of solitary laughter behaviors enable us to begin to disentangle this complex phenomenon. Solitary laughter deserves more research not only to reduce the stigma often associated with it, but also to better address related pathologies, and to unleash its potential therapeutic benefits.

## Supplementary Information

Below is the link to the electronic supplementary material.

Supplementary file1 (PDF 180 KB)

## Data Availability

No datasets were generated or analysed during the current study.
