# Supplementary material for: Liberating the mental health and wellbeing benefits of laughing alone: a new taxonomic model and scoping review for future research
Source: Discov Ment Health. 2025 Apr 28;5(1):63. doi: 10.1007/s44192-025-00183-9 (PMC12037970; doi:10.1007/s44192-025-00183-9)
Supplement: Supplementary file 1 — Supplementary file1 (PDF 180 KB) [file 44192_2025_183_MOESM1_ESM.pdf]

## APPENDIX

**Appendix Table:** Selected solitary laughter quotations from literary and other works (*n* = 37)

(Note. Bracketed dates are the believed original dates of publication.)

| **Reference** | **Quotation** | **Possible Interpretation SL=Solitary Laughter** |
|---|---|---|
| Augustine, 1909 [401] | *"Because none doth ordinarily laugh alone? ordinarily no one; yet laughter sometimes masters men alone and singly when no one whatever is with them, if anything very ludicrous presents itself to their senses or mind. Yet I had not done this alone; alone I had never done it."* | SL occurs from comical thoughts (though deemed morally dangerous). |
| Beck, 2008 | *"I laugh alone at the idea that Marilyn Monroe might whisper, 'Eat me'..."* | SL elicited by an incongruous image. |
| Ben-Moshe, 2017 | *"We need to recondition our belief systems to empower ourselves to laugh alone and for our own sake."* | A change in thinking about SL is needed. |
| Ben-Moshe & Gonot-Schoupinsky, 2024 | *"If you're a self*-conscious *laugher, laugh in places you feel less vulnerable: the shower, home alone, or in the car… ."* | SL is a way to enjoy laughter without feeling vulnerable. |
| Bergson, 1914 [1900] | *"To understand laughter, we must put it back into its natural environment, which is society, and above all must we determine the utility of its function, which is a social one. ... Laughter must answer to certain requirements of life in common. It must have a social signification."* | Laughter must be interpreted socially. |
| Bushnell, 2006 | *"Mom, watching Letterman, laughing alone in the empty apartment..."* | SL alone with media is viewed from outside. |
| Casanova, 1913 [18th c.] | *"I give myself over often to outbursts of laughter which makes me pass for a fool as idiots do not believe that one can laugh alone."* | Pleasure in laughing while writing. |
| Congreve, 1711 [1694] | *"But there is nothing more unbecoming a Man of Quality than to Laugh... such a Vulgar Expression.... Every body can Laugh... To be pleased with what pleases the Crowd. Now when I Laugh, I always Laugh alone."* (Froth) | Laughing alone preferred to public laughter. |
| Cox & Ince, 2020 | *"Often when I'm writing comic material, I will laugh at the joke I've just written.... So, I am sort of tickling myself."* (Frank Skinner) | Creating comedy alone can provoke SL. |

| | | |
|---|---|---|
| Daniels, 2007 | *"It makes me laugh, alone in bed..."* | SL in relation to thoughts of the cosmic/divine. |
| Davis, 1993 | *"Even most seemingly solitary laughter is actually 'pseudosocial,' requiring a split self or imagined others."* | SL is achieved by split self or imagined others. |
| Dawkins, 1960 | *"Laughing alone calls attention to oneself."* | SL as attention-seeking. |
| Earleywine, 2010 | *"Solo laughter often entails imagined social interaction—we envision others talking or listening as we chuckle on our own"* | SL requires imagined others. |
| Forster, 1962 [1907] | *"One day you were laughing alone all down in the sweet peas."* | A character laughing alone outside. |
| Freud, 1960 [1905] | *"...one can enjoy the comic alone... I can laugh heartily over it alone; I am naturally pleased if by imparting it to someone else I make him laugh too."* | Enjoying SL is possible, though sharing can also be pleasurable. |
| Gibran, 1963 [1918] | *"... in my heart I laughed at thy love. Yet I would not have thee see my laughter. I would laugh alone."* | Keeping laughter to oneself to avoid hurt to another. |
| Gold, 2008 | *"I've learned to go to the movies and to hike alone; it's harder to learn to laugh alone."* | SL is a difficult behavior to acquire. |
| Gonot-Schoupinsky et al., 2024 | *"Solirisy (laughing alone, or solitary laughter) can be practical, enjoyable, and beneficial."* | SL can be beneficial. |
| Halliwell, 2005 | *"... the Abderites invite Hippocrates to treat their fellow-citizen, whose continual (and, significantly, solitary) laughter makes them think him mad."* | SL and madness in Ancient Greece. |
| Harmon, 2007 | *"I found myself laughing, all alone in the dark, I made myself laugh, I laughed to myself."* | Laughing at one's own comparisons, incongruity. |
| Hawthorne, 1974 [1852] | *"... this strange man looked inward at his own heart, and burst into laughter that rolled away into the night."* | SL as something that is frightening. |
| Kierkegaard 1944 [1843] | *"...the devil is also said to laugh when he is alone."... "I do not ask whether it sometimes or even frequently occurs to you to laugh when you are alone, but whether you take satisfaction in this lonely laughter."* | SL is associated with the devil, and considered to reflect a limited, depressing existence. |
| Laertius,1980 [3rd c.] | *"[Myson of Chenae] was seen in Lacedaemon laughing to himself in a lonely spot; and when someone suddenly* | Reveals that solitude may induce SL. |

| | | |
|---|---|---|
| | *appeared and asked him why he laughed when no one was near, he replied, 'That is just the reason'."* | |
| Mayne, 1888 [1829] | *"If I weep, I weep; if I laugh, I must laugh alone"* | Certain laughter cannot be shared. |
| de Montaigne, 1877 [1580] | *"... we must for the most part entertain ourselves with ourselves, and so privately that no exotic knowledge or communication be admitted there; there to laugh and to talk, as if without wife, children, goods, train, or attendance..."* | Eventually, we will be alone, so why not enjoy laughing alone now? |
| Nelson, 2021 | *"A few times in my life I have awaked/Laughing out loud at a wonderful joke./I never recall the joke, but keep on laughing/For a minute or two, alone in the dark./A few nights ago my friend Jacques said something/That laughed me awake, still laughing at Jacques,/Then laughing at laughing myself awake by laughing,/With no idea of what could have been so funny./I lay there smiling and musing, wondering/If death is the punch line, and life the joke./Or vice versa. Quack, quack."* | SL initially induced by memory and then became self-sustaining, finally led to existential questioning. |
| Nietzsche, 1996 [1878] | *"Some people are so used to solitude with themselves that they never compare themselves to others, but spin forth their monologue of a life in a calm, joyous mood, holding good conversations with themselves, even laughing."* | SL can be enjoyed when one knows how to do it. |
| Palahniuk, 1996 | *"Panicking by yourself is like laughing alone in an empty room. Even if you're watching t.v., you feel really silly."* | Laughing alone may be embarrassing, even with media. |
| Schopenhauer, 2004 [1851] | *"I am not surprised that some people are bored when they find themselves alone; for they cannot laugh if they are quite by themselves. The very idea of it seems folly to them. Are we, then, to look upon laughter as merely a signal for others—a mere sign, like a word? What makes it impossible for people to laugh when they are alone is nothing but want of imagination, dullness of mind."* | Those who are bored in solitary confinement lack the ability to imagine and thereby laugh alone. |
| Shakespeare, 2009 [1597] | *"Now will he sit under a medlar tree,/And wish his mistress were that kind of fruit/As maids call medlars when they laugh alone."* (Mercutio) | Inference: we are freed to laugh at innuendo when alone. (To meddle was to masturbate.) |
| Sheppard, 1975 | *".. words like... boffin and horripilating ... have caused two generations of grown men with attaché cases to break up in solitary laughter on public transport."* | Highlights how sometimes laughing alone is very acceptable. |
| Steinberg, 2002 | *"I would shake my head, say, That leaky faucet, and laugh alone."* | Laughing alone to deal emotionally with a situation. |

| | | |
|---|---|---|
| Tolstoy, 1968 [1869] | *“Men looked up in astonishment at the sound of this curious and evidently solitary laughter."* | Wonder at good-natured solitary laughter in company. |
| Warren, 1976 | *“Laughing alone with a sound like a strangled loon-call,...”* | Laughing alone is poetically associated with natural sounds. |
| Webster, 1870 | *“Pshaw! The trash is savourless today: one cannot laugh alone.”* | Laughing alone is not possible. |
| Wister, 1921 [1906] | *“The sudden thought of this made me laugh alone in my bed of sickness.”* | Laughter from thought. |
| Wordsworth, 2018 [1814] | *“... to laugh alone,/At a composing distance from the haunts/Of strife and folly, though it be a treat/As choice as musing Leisure can bestow;”* | Wordsworth describes pleasure in disengaged solitary laughter. |

**Table References**


Augustine, Saint (1909). *The Confessions of Saint Augustine*. Project Gutenberg eBook.

Beck, S. (2008). Cue the squirt. *Fourth Genre: Explorations in Nonfiction, 10*(1), 95-104.

Ben-Moshe, R. (2017). *Laughing at Cancer: How to Heal with Love, Laughter and Mindfulness*. Brolga.

Ben-Moshe, R., & Gonot-Schoupinsky, F. (2024). Laughter, mental health and cancer: a case study of Ros Ben-Moshe. *Mental Health and Social Inclusion, 28*(5), 448-459.

Bergson, H., Brereton, C. S. H., & Rothwell, F. (1914). *Laughter: An essay on the Meaning of the Comic*. Macmillan.

Bushnell, J. T. (2006). Amanda said I should go to Cuba. *Mississippi Review, 34*(1/2), 23-36.

Casanova: G. *Correspondance avec J. F. Ofiz, Leipzig*, 1913, I, p.80. Quoted in Childs, J. R. (1954). Further clues bearing on the mystery of Casanova's "Memoirs." *The Papers of the Bibliographical Society of America, 48*(3), 248-262.

Congreve, W. (1711). *The Double-dealer: A Comedy*. T. Johnson.

Cox, B. & Ince, R. (2020). Science of laughter, *The Infinite Monkey Cage*. BBC.

Daniels, J. (2007). *Literary Review,* 5(2), 79-83.

Davis, M. S. (1993). *What's so funny?: The Comic Conception of Culture and Society*. University of Chicago Press.

Dawkins, C. (1960). The Quiet Enemy. *The Sewanee Review, 68*(1), 61-88.

Earleywine, M. (2010). *Humor 101*. Springer.

Forster, E. M. (1962). *The Longest Journey*. New York: Vintage Press.

Freud, S. (1960). *Jokes and Their Relation to the Unconscious*. WW Norton & Company.

Gibran K. (1963). *The Madman: His Parables and Poems* (London, Heinemann).

Gold, H. (2008). *Still Alive!: A Temporary Condition: a Memoir.* Arcade.

Gonot-Schoupinsky, F., Neal, M., & Carson, J. (2024). *The Positive Psychology of Laughter and Humour*. Emerald Publishing.

Gonot-Schoupinsky, F., Neal, M., & Carson, J. (2024). The Future of Laughter and Humour in Positive Psychology. *The Positive Psychology of Laughter and Humour* (pp. 117-125). Emerald Publishing.

Halliwell, S. (2005). Greek Laughter and the Problem of the Absurd. *Arion: A Journal of Humanities and the Classics, 13*(2), 121-146.

Harmon, L. (2007). Illuminating the Dark: The Stories of Lowell B. Komie and the Pursuit of Meaningful Work. *Legal Stud. F., 31*, 851.

Hawthorne, N. (1974). Ethan Brand: A Chapter from an Abortive Romance. *The Centenary Edition of the Works of Nathaniel Hawthorne*, 11, 82-102.

Kierkegaard, Soren. (1944). *Either/Or II.* Oxford University Press.

Laertius, D. (1980). *Lives of Eminent Philosophers.* Harvard University Press.

Mayne, E. (1888). TCM. Letters of Rev. Edward F. Mayne, 1829-1834 5(4). *The American Catholic Historical Researches*, 184-187.

De Montaine, M. (1877). *The Essays of Montaigne, Complete by Michel de Montaigne*. Project Gutenberg eBook.

Nelson, M. (2021). The Punch Line. *The Hudson Review, 73*(4).

Nietzsche, F. (1996). *Human, all too Human*. Cambridge University Press.

Palahniuk, C. (1996). Invisible Monsters. *Columbia: A Journal of Literature and Art, 26*, 8-17.

Schopenhauer, A. (2004). *Essays of Schopenhauer*. Project Gutenberg eBook.

Shakespeare, W. (2009). *The Tragedy of Romeo and Juliet*. Project Gutenberg eBook.

Sheppard, R. Z. (1975). Review of Vinegar Puss by S.J. Perelman. *TIME Magazine, 105*(14), 76-77.

Steinberg, S. & Steingberg, S. (2002). Caught. *Columbia: A Journal of Literature and Art, 36*, 99-106.

Tolstoy, L. (1968) *War and Peace*. New American Library,

Warren, R. P. (1976). Sister Water. *The New York Review* (Online). October 14, 1976.

Webster, A. (1870). *A Castaway in Portraits*. MacMillan.

Wister, O. (1921). *Lady Baltimore.* Macmillan.

Wordsworth (1814). The Excursion: Book Third. *The Poetical Works of William Wordsworth Vol.5*. Macmillan.
